# Risk Factors and Protection Associated with Well-Being and Psychological Distress of Veterinarians in Brazil

**DOI:** 10.3390/vetsci12090835

**Published:** 2025-08-29

**Authors:** Bianca S. Gresele, Jefferson L. Pereira, Anderson da S. Rosa, Helena C. Lyrio-Carvalho, Sofia M. V. Ulisses, Alexandre R. S. da Silva

**Affiliations:** 1 Graduate Program in Clinical Psychology, Department of Clinical Psychology, Pontifícia Universidade Católica de São Paulo (PUC), São Paulo 05015-901, Brazil; ra00192857@pucsp.edu.br (J.L.P.); hclcarvalho@uol.com.br (H.C.L.-C.); 2 Collective Health Department, Escola Paulista de Enfermagem, Universidade Federal de São Paulo (UNIFESP), São Paulo 04024-002, Brazil; anderson.rosa@unifesp.br; 3 School of Education, University of California, Riverside, CA 92521, USA; smarq039@ucr.edu; 4 Graduate Program in Veterinary Sciences in the Semi-arid Region and College of Veterinary Medicine, Agricultural Sciences Campus, Universidade Federal do Vale do São Francisco (UNIVASF), Petrolina 56304-917, Brazil; alexandre.redson@univasf.edu.br

**Keywords:** veterinarians, psychological distress, mental health, coping skills, well-being, occupational stress, protective factors

## Abstract

Veterinary professionals are exposed to intense emotional and occupational demands that can compromise mental health. This nationwide cross-sectional study surveyed 1992 Brazilian veterinarians to assess their psychological distress, well-being, and coping strategies. Logistic regression identified that women, early-career professionals, and individuals with low income were more likely to experience psychological distress. Long working hours and professional dissatisfaction were significant risk factors. In contrast, adaptive coping behaviors, such as spending time with the family, sleeping at least eight hours per night, and engaging in leisure activities, were associated with better mental health outcomes. Institutional wellness programs tailored to the veterinary profession should incorporate strategies to mitigate stress and promote resilience. Although the cross-sectional design limits causal inference, the large sample size and use of validated measures provide robust insights. Future research should adopt longitudinal and interventional designs to refine the strategies that support veterinarians’ mental well-being.

## Simple Summary

This study investigated psychological distress and well-being among 1992 Brazilian veterinarians using validated instruments (K6, PWBI). A high prevalence of distress was found, especially among women, younger professionals, and those with lower income. Longer working hours, adverse conditions, and career dissatisfaction were identified as risk factors. On the other hand, family support, sufficient sleep, and leisure activities emerged as protective factors against psychological distress. These findings highlight the need for institutional mental health policies, particularly for early-career professionals. Promoting adaptive coping strategies and fostering work–life balance may enhance well-being in this high-demand profession.

## 1. Introduction

The mental health of healthcare professionals has been widely studied because of its critical role in both individual and collective well-being, as well as its direct impact on the quality of services provided to the population. Among these professionals, veterinarians are considered particularly vulnerable to psychological distress due to the unique challenges of their work, which include animal care, client interactions, and complex ethical decisions [1,2,3]. In the case of pets, emotional demands may involve supporting clients during an animal’s illness, coping with the veterinarian’s own feelings of grief, managing client grief after an animal’s death, and addressing the ethical dilemmas surrounding euthanasia [1,2,3,4]. In this context, veterinarians’ mental health has become a significant concern, with growing evidence of high stress levels, fatigue, and impaired well-being [5,6,7]. Studies conducted in the United Kingdom [8], the United States [5,9], Australia [10], and Canada [11] have reported that veterinarians experience heavy emotional workloads and notable professional dissatisfaction, underscoring the urgency of interventions aimed at promoting their psychological well-being.

Although psychological distress is widely documented in several health areas, Brazil lacks national research that specifically investigates the mental health of veterinarians, highlighting a significant gap in the understanding of the challenges faced by these professionals. This scenario is particularly concerning given that Brazil has one of the highest per capita concentrations of veterinarians worldwide. By 2022, Brazil had an average of 77.4 veterinarians per 100,000 inhabitants, with approximately 9250 new professionals entering the profession every year, which is lower than in Latvia, a country with 1,934,379 inhabitants and 2500 veterinarians, yielding a ratio of 129 professionals per 100,000 inhabitants [12]. Brazil has more than 200,000 registered veterinarians, with a predominance of female veterinarians in clinical practice. Most professionals work in the private sector, particularly in companion animal clinics, and there is considerable regional heterogeneity in working conditions due to the country’s continental dimensions [12]. The Brazilian veterinary sector is characterized by high professional demands, regional inequalities, and limited mental health infrastructure, making it particularly vulnerable to the challenges addressed in this study [12].

The literature shows that sociodemographic factors, including age, gender, and education, influence workers’ mental health across several fields. For example, young women and early-career professionals frequently report greater vulnerability to psychological distress [13,14]. In addition, employment type, workload, and balance between personal and professional life also play essential roles in the well-being of these professionals [15,16,17]. However, the relationship between these factors and mental health outcomes has been poorly explored in the context of veterinary medicine, particularly in representative samples [18,19].

Another key aspect for understanding the factors involved in the mental health of veterinary professionals is the coping strategies they use to manage stressful situations in the workplace. This study is grounded in the stress-coping framework proposed by Lazarus and Folkman [20], which defines coping as a set of strategies used to address emotional and situational challenges. These strategies can be adaptive or maladaptive depending on the effectiveness of the response [20]. Adaptive coping strategies, such as seeking social support and positive reassessment, have been associated with better mental health outcomes [21,22]. However, few studies have examined how sociodemographic and occupational factors interact with coping strategies in the veterinary context.

Using a representative national sample, this study explored how sociodemographic and occupational factors, along with coping strategies, interact to predict the mental health of Brazilian veterinarians, specifically in terms of well-being and psychological distress. This research was inspired by a study conducted in the United States by Merck and replicated in Brazil with support from Merck Sharp & Dohme São Paulo, Brazil (MSD Brazil) [7,23]. While previous studies have consistently documented high levels of stress among veterinarians, findings on the protective role of coping strategies and the influence of demographic factors remain inconclusive. For instance, although physical activity is widely considered beneficial, some studies have reported mixed associations with psychological outcomes in this population [24,25].

This study sought to determine which factors significantly influence veterinarians’ mental health, and how these factors interact. Notably, the findings revealed that career dissatisfaction, excessive workload, and poor work–life balance were central to psychological distress, whereas adaptive coping behaviors and social support were associated with preserved well-being. These insights are critical for informing targeted interventions and institutional policies that foster sustainable and mentally healthy careers in veterinary medicine.

Psychological distress, in the context of this research, is defined as the presence of symptoms related to anxiety and depression that impair emotional functioning, as assessed by the Kessler Psychological Distress Scale (K6) [26]. Well-being refers to the self-perceived experience of emotional balance and functional vitality, as evaluated by the Physician Well-Being Index (PWBI), which captures emotional exhaustion, negative mood states, perceived stress, and functional impairment related to mental and physical health [27]. These two constructs are considered distinct yet complementary dimensions of mental health.

Accordingly, the present study aimed to investigate how sociodemographic characteristics, occupational conditions, and coping strategies contribute to the psychological distress and well-being among Brazilian veterinarians, drawing on a representative national sample.

## 2. Materials and Methods

### 2.1. Study Design and Participants

A cross-sectional study was conducted between 29 June and 25 July 2022, involving male and female veterinarians actively working in Brazil. This study was approved by the Human Research Ethics Committee of the Faculdade Inspirar (Opinion No. 31645220.4.0000.5594) and was registered with the National Research Ethics Commission (CONEP) of the Brazilian Ministry of Health.

Participants were recruited via email from registry data provided by the National Association of Veterinary Clinicians for Small Animals (Anclivepa Brazil) and MSD Brazil, comprising approximately 20,000 registered professionals. Inclusion criteria required participants to be of legal age (≥18 years), corresponding to adulthood in Brazil. As veterinary medicine in Brazil is a post-secondary program requiring a high school diploma, all participants had completed at least some undergraduate level of education. Approximately 2000 professionals completed the online questionnaire, and 1992 responses were deemed valid for analysis. A convenience sampling strategy was used based on existing professional registries, which may have introduced selection bias.

Participation was voluntary and secured following the presentation of the study objectives and the completion of the Informed Consent Form, with anonymity guaranteed throughout all stages of the study. The participants worked in clinical veterinary practice across different regions of Brazil.

Although recruitment efforts aimed to ensure geographic and sectoral diversity, convenience sampling may have produced a sample that is not fully representative of the broader veterinary population. In particular, individuals who were more aware of or concerned with mental health topics may have been more inclined to participate. Thus, caution is warranted when generalizing these findings to all Brazilian veterinarians.

### 2.2. Instruments

This study collected data on participants’ sociodemographic characteristics, including age, gender, ethnicity, marital status, number of children, and type of residence. Occupational information included income, professional position, years since graduation, weekly workload, secondary employment, and level of satisfaction with the profession. Coping strategies were selected based on the prior literature identifying common self-care behaviors among healthcare professionals and national wellness surveys in the veterinary field.

Coping strategies were assessed using ten questions on a four-point Likert scale (frequently, sometimes, seldom, and never). The activities analyzed included spending time with family, engaging in physical exercise, sleeping at least eight hours per night, socializing with friends, dedicating time to hobbies, reading for pleasure, traveling for leisure, volunteering, and practicing yoga. The total score demonstrated internal consistency, with a coefficient alpha of 0.75, which is considered acceptable for exploratory research [28].

Psychological distress was measured using the Kessler Screening Scale for Psychological Distress (K6) [26]. The validated Brazilian version of the K6 demonstrates good psychometric properties, including correlations with clinical diagnoses and other psychological scales [29]. The K6 comprises six questions assessing the frequency with which individuals have experienced symptoms of anxiety and depression over the past 30 days. Responses are rated on a five-point Likert scale from 0 (none of the time) to 4 (all the time), leading to a total score ranging from 0 to 24. A score of 13 or above (K6 ≥ 13) [26] indicates a high probability of psychological distress. The scale demonstrated acceptable internal consistency, with a coefficient alpha of 0.81 [28].

Well-being was assessed using the Physician Well-Being Index (PWBI) [27]. The PWBI consists of seven binary items (0 = No; 1 = Yes) assessing exhaustion, discouragement, negative perceptions of quality of life, stress, fatigue, and other indicators of emotional strain. Scores are calculated by summing all affirmative responses, with higher scores indicating impaired well-being. A cutoff of <4 was adopted to indicate preserved well-being. For regression analyses, the PWBI was dichotomized according to this cutoff (<4 = preserved; ≥4 = impaired) and then recoded so that preserved well-being was coded as 1 and impaired well-being as 0 (see statistical analysis). The total PWBI score showed evidence of internal consistency, with an alpha coefficient of 0.72. Evidence of validity includes associations with indicators of burnout, distress, and professional performance in previous studies [27].

The K6 and PWBI were selected for their brevity, robust psychometric properties, and prior use in occupational mental health studies, making them appropriate for busy professionals, such as veterinarians [26,27,29].

### 2.3. Statistical Analysis

Sample characteristics and descriptions are presented as frequencies (n) and percentages, respectively. K6 results are reported as mean, standard deviation, and 95% confidence interval (CI). PWBI results are presented as frequency (n) and percentage of the total sample. Responses of “I prefer not to answer” were treated as missing values. In this study, the proportion of missing values for variables ranged up to 10% across variables; therefore, all missing values were entered using the Expected Maximization (EM) algorithm method. The EM algorithm was chosen due to its robust performance in handling datasets with up to 10% missing data. Compared to simpler techniques such as listwise deletion or mean substitution, EM preserves statistical power and reduces bias in parameter estimates, which is particularly important in large-scale surveys where maintaining data completeness is essential for reliable multivariate analyses.

Multiple logistic regression was used to assess associations between sociodemographic factors, occupational factors, and coping strategies with the categories of K6 (≥13 = possible presence of psychological distress; <13 = absence) and PWBI (≥4 = impaired well-being; <4 = preserved well-being). Given the binary nature of both outcomes, logistic regression enabled the estimation of adjusted odds ratios. For consistency in interpretation, the PWBI variable was recoded so that higher values indicated preserved well-being (0 = impaired, 1 = preserved). This approach allowed direct comparison of odds ratios across the two mental health indicators. The assumptions of multicollinearity were verified using tolerance and variance inflation factors, and the linearity of the continuous variables was verified using the Box–Tidwell transformation. The quality of the models was assessed using the Omnibus Test of Model, R2 Nagelkerke, and Hosmer–Lemeshow tests. Statistical analyses were performed using IBM SPSS Statistics software (version 27). The a priori probability of type-I error was set at 5%.

## 3. Results

### 3.1. Sociodemographic, Occupational, and Mental Health Outcomes

In terms of sociodemographic data, 78% of participants were women (1545/1992), 22% were men (434/1992), and 1% (13/1992) identified with other genders. The mean age of participants was 37.1 ± 10.02 years (95% CI = 36.7–37.5). Most participants identified as white (75%, 1486/1992), followed by brown (19%, 374/1992), black (3%, 67/1992), yellow (2%, 33/1992), and other (1%, 13/1992). In terms of marital status, 52% (1026/1992) were married or in a stable union, while 41% (821/1992) were single. In addition, 60% (1191/1992) reported not having children. Geographically, most participants were located in the Southeast (59%), followed by the South (20%), Northeast (12%), and North (3%).

Regarding employment data (Table 1), 63% (1261/1992) of respondents reported earning up to USD 1165 per month, while 26% (522/1992) reported earning USD 1166 or more. The most common positions were veterinary clinic employees (37%), veterinary clinic owners (28%), and surrogate veterinarians (13%). Among all respondents, 74% (1472 of 1992) reported clinical experience. The majority (58%) had been practicing the profession for up to ten years, while 42% (841/1992) had at least 11 years of professional experience. Regarding workload, nearly half the participants reported working more hours than desired, while the remainder indicated either satisfaction with their workload or that they worked fewer hours than preferred. Additionally, 39% reported holding a second job. Regarding job satisfaction, 57% (1126/1992) of the respondents stated that they would not recommend a career in veterinary medicine, and 27% (542/1992) reported regretting their professional choice.

**Figure 1:**
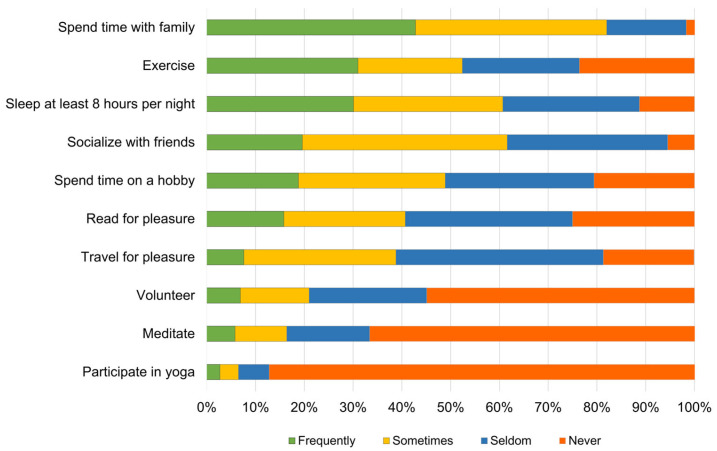
Coping strategies reported by participants (n = 1992).

As shown in Figure 1, the most frequently reported coping strategies were spending time with family (43%), exercising (31%), and sleeping for at least eight hours per night (30%).

The K6 scale indicated an average psychological distress score of 10.35 (SD = 5.019, 95% CI = 10.13–10.57), with 654 participants (33%) classified as experiencing psychological distress. For the PWBI, the average score was 4.23 (SD = 1.85; 95% CI = 4.15–4.31), with 29% (586/1992) of the participants scoring up to three points, indicating preserved well-being.

### 3.2. Sociodemographic and Occupational Factors as Predictors of Mental Health Outcomes

Table 2 presents the results of the multiple logistic regression analysis, which examined the relationship between psychological distress assessed by the K6, well-being assessed by the PWBI, and sociodemographic and occupational factors. Ethnicity, macro-region, and profession role were excluded from the model, as the literature provides no consistent evidence that these variables differentiate levels of psychological distress or well-being.

The findings from the K6 scale indicate that compared with single individuals, married participants had 26% lower odds of reporting psychological distress (OR = 0.74, 95% CI = 0.59–0.92), suggesting that marriage may act as a protective factor, likely by providing emotional support and shared responsibilities that buffer occupational stress. Higher income was also associated with lower odds of psychological distress (OR = 0.85; 95% CI = 0.75–0.98). In addition, more years since graduation were associated with reduced risk, with each additional year linked to 2% lower odds of distress (OR = 0.98; 95% CI = 0.95–0.99).

Participants who reported working more hours than desired had 74% higher odds of experiencing psychological distress compared with those satisfied with their workload (OR = 1.74, 95% CI = 1.37–2.21). In addition, career-related dissatisfaction was strongly associated with distress: respondents who would not recommend a veterinary career had 84% higher odds (OR = 1.84, 95% CI = 1.42–2.35), and those who regretted choosing the profession had 153% higher odds (OR = 2.53, 95% CI = 2.00–3.20) of experiencing psychological distress.

As shown in Table 2, the analysis also examined sociodemographic predictors of well-being (PWBI). Remarkably, participants who reported working more hours than desired had 69% lower odds of preserved well-being (OR = 0.31, 95% CI = 0.24–0.40). Age, sex, marital status, and second job status were also significant. Notably, female professionals had 52% lower odds of preserved well-being than males (OR = 0.48; 95% CI = 0.37–0.62), which may reflect the cumulative effects of gender-based disparities, including wage inequality, caregiving responsibilities, and expectations for emotional labor in clinical settings. In addition, those who would not recommend veterinary medicine as a career had 58% lower odds of preserved well-being (OR = 0.42; 95% CI = 0.32–0.53), whereas those who regretted entering the profession had 46% lower odds (OR = 0.54; 95% CI = 0.39–0.74).

### 3.3. Coping Strategies as Predictors of Mental Health Outcomes

Table 3 presents the results of multiple binary logistic regression analysis, examining the association between coping strategies, psychological distress, and well-being. Overall, more frequent engagement in coping-related activities was associated with lower psychological distress and preserved well-being. When considering predictors of psychological distress measured by the K6, several activities emerged as significant protective factors, ordered from strongest to weakest association: traveling for pleasure (OR = 0.67, 95% CI = 0.58–0.78), spending time with family (OR = 0.69, 95% CI = 0.60–0.80), sleeping at least eight hours a night (OR = 0.80, 95% CI = 0.72–0.89), engaging in hobbies (OR = 0.81, 95% CI = 0.71–0.92), socializing with friends (OR = 0.82, 95% CI = 0.71–0.95), and participating in volunteer work (OR = 0.86, 95% CI = 0.76–0.97).

When the impact of coping strategies on well-being (PWBI) was examined using multiple logistic regression (Table 3), 7 of the 11 activities remained significant. Traveling for pleasure and sleeping at least eight hours per night were associated with 36% higher odds of preserved well-being (OR = 1.36, 95% CI = 1.17–1.57, and OR = 1.36, 95% CI = 1.21–1.53, respectively). In contrast, practicing yoga was associated with 21% lower odds of preserved well-being (OR = 0.79, 95% CI = 0.66–0.94), which contrasts with previous findings [30,31].

Although several coping strategies demonstrated statistical significance, it is important to consider their practical implications. For instance, spending time with family (OR = 0.69, 95% CI = 0.60–0.80) and traveling for pleasure (OR = 0.67) were associated with approximately 30–33% lower odds of psychological distress—effects that are not only statistically significant but potentially clinically meaningful. These insights may inform simple, low-cost interventions in wellness programs for veterinary professionals.

## 4. Discussion

This study investigated the key factors influencing veterinarians’ mental health and how these factors interact. The findings revealed that dissatisfaction with career choice, excessive workload, and lack of work–life balance were central to psychological distress, whereas adaptive behaviors and social support were linked to preserved well-being. These insights are critical for informing targeted interventions and institutional policies that foster sustainable and mentally healthy veterinary medicine careers.

Among Brazilian veterinarians, 32.8% scored high on the K6, revealing a high prevalence of psychological distress. These findings are consistent with international studies reporting high levels of psychological distress among veterinary medicine professionals [7,32,33]. Veterinary practice entails distinct challenges, including euthanasia, managing animal suffering, navigating ethical and moral dilemmas, handling excessive workload, and addressing pet owners’ emotional needs. Such factors can substantially contribute to occupational stress and mental health risks [14].

Among the sociodemographic factors analyzed, female veterinarians were more vulnerable to psychological distress and reported impaired well-being compared with males. This finding aligns with the existing literature, identifying women as being more susceptible to psychological distress in veterinary medicine, often due to structural inequalities, double shifts, and higher exposure to emotionally demanding tasks [34,35]. In addition, married individuals exhibited lower odds of experiencing psychological distress compared with single individuals, likely reflecting the protective role of social support, which has been widely recognized across occupational contexts [8,20].

Lower income levels were associated with higher psychological distress, underscoring the significant impact of financial stress on mental health within the profession, a finding consistent with previous research [7,23]. Early-career veterinarians reported higher distress and impaired well-being when compared to their more experienced counterparts, suggesting that early-career veterinarians are particularly vulnerable to emotional difficulties. This result echoes prior evidence of worsening mental health among veterinary students and early-career practitioners (18–34 years).

Although these findings mirror international concerns, notable differences exist. For instance, in North America and Europe, financial debt and reduced professional autonomy are frequently cited as primary stressors [7]. In contrast, Brazilian veterinarians in this study highlighted work overload and career dissatisfaction as predominant concerns. These differences may reflect systemic and cultural variations in veterinary practices, healthcare infrastructure, and social expectations across countries.

One of the most significant findings of this study was the strong association between working hours and psychological distress, a relationship widely documented in the international literature [6,10,18,33,35]. In line with these findings, this study demonstrated that adverse working conditions were strongly associated with psychological distress among Brazilian veterinarians.

The data of this study indicate a concerning level of dissatisfaction among veterinary medicine professionals in Brazil. More than half of participants (57%) reported that they would not recommend the profession, and 27% regretted their career choice. These results are consistent with studies showing increasing dissatisfaction among veterinarians worldwide [36,37]. In addition, they reflect similar trends noted in studies conducted in the United States, in which 52% of veterinarians indicated that they would not recommend the profession [14,23]. Such dissatisfaction has often been attributed to work overload, low remuneration, and unmet professional expectations [6,10]. The imbalance between professional and personal lives also appears in multiple studies as one of the main predictors of psychological distress [5,38]. Importantly, European studies have also highlighted burnout as a related outcome, frequently arising from the same stressors observed in our sample [6,19,33,35,39,40]. Although our study did not directly assess burnout, the overlap with dissatisfaction and impaired well-being suggests that burnout may represent an additional dimension of psychological burden among Brazilian veterinarians, warranting attention in future research and interventions.

Coping strategies have been shown to be a significant protective factor against psychological distress. Activities such as traveling for leisure, spending time with family (the most frequently reported practice), sleeping at least eight hours a night, practicing hobbies, socializing with friends, and volunteering were significantly associated with reduced psychological distress and preserved well-being. In contrast, practicing yoga showed a negative association with preserved well-being. This result may reflect reverse causality—individuals already experiencing distress may be more likely to engage in yoga as a coping attempt—or may involve contextual or confounding factors not captured in this study. These findings reinforce classical theories on coping, and recent evidence indicates that the use of adaptive coping strategies is effective in preventing psychological distress among health professionals [41,42,43,44].

These results suggest that institutional policies should prioritize work-hour regulation, especially for early-career professionals. Potential interventions include mentorship programs, mental health literacy training, and institutional protocols for ensuring adequate rest. Promoting professional networks and peer support groups may be effective alternatives for self-employed professionals. These interventions must be adapted to the diverse realities of employment in veterinary medicine.

However, it is worth emphasizing that, despite extensive evidence associating physical activity with reduced psychological distress [45,46], this study found no significant association between physical exercise and either K6 or PWBI scores. Recent systematic reviews further confirm that regular physical activity is consistently linked to lower risk of depression, anxiety, and burnout across health professionals and the general population [47,48]. This lack of association found in this study contrasts with much of the existing literature and may be explained by several factors. First, the measurement of physical activity was based on self-report and did not capture essential dimensions such as frequency, intensity, duration, or type of exercise, which are known to influence mental health outcomes differently. Second, contextual and occupational stressors specific to veterinary medicine—including excessive workload, irregular schedules, exposure to animal suffering, and emotionally demanding interactions with clients—may attenuate or even outweigh the potential protective effects of exercise. Third, it is possible that the benefits of physical activity are more evident in populations with lower baseline levels of occupational stress or in longitudinal and interventional designs, while cross-sectional studies, such as the present one, may fail to capture cumulative or long-term effects. Moreover, the interaction of physical activity with other coping strategies, such as social support, adequate sleep, or leisure activities, may be necessary for its protective effects to manifest. In this sense, physical activity alone may not be sufficient to mitigate psychological burden in highly demanding professional contexts. These findings underscore the complexity of the relationship between exercise and mental health and highlight the need for further investigations using more rigorous methodologies—such as randomized clinical trials—to establish causal relationships and to clarify whether the presumed benefits of physical activity, yoga, or other lifestyle interventions are consistently observed across different populations and occupational settings.

This study is not without limitations. As a cross-sectional design, it precludes causal inferences. Furthermore, the use of non-probabilistic sampling and reliance on volunteer participation may have introduced a selection bias, potentially favoring individuals more aware of or interested in mental health, thereby limiting generalizability. Future research should incorporate mixed methods, including qualitative and longitudinal approaches, to better capture the complex interplay of sociodemographic and occupational factors, coping strategies, and mental health in the veterinary profession. Reliance on self-reported data may also have introduced social desirability biases, potentially leading to the underreporting of negative mental health experiences. In addition, potential confounding variables such as personality traits or access to mental health services were not assessed and may have influenced the outcomes. The absence of clinical assessments or objective indicators (e.g., health records and workload metrics) limits the validation of reported symptoms and coping behaviors. Finally, variables such as ethnicity and geographic region were not included in the regression models due to sample distribution and statistical power constraints. Nonetheless, these factors are highly relevant in the Brazilian context, and future studies should explore their influence on mental health outcomes more thoroughly.

## 5. Conclusions

This study revealed a high prevalence of psychological distress among Brazilian veterinarians, which was strongly associated with work overload, career dissatisfaction, and low-income status. Protective factors such as adaptive coping behaviors, strong social support networks, and satisfaction with work–life balance were linked to preserved well-being. Early-career professionals, women, and those with lower income levels were particularly vulnerable. These findings highlight the need for mental health support strategies tailored to the specific working conditions of veterinarians. For those in institutional settings, wellness programs should promote coping strategies that directly address occupational stressors, such as flexible scheduling, planned rest periods, and encouragement of leisure and family time. For self-employed professionals and clinic owners, relevant strategies may include clearer boundaries between personal and professional life, proactive scheduling of time off, delegation of administrative tasks, and prioritization of restorative practices such as regular sleep, hobbies, and time with loved ones. Regardless of work context, coping strategies associated with reduced distress in this study—such as sleeping at least eight hours per night, engaging in leisure travel, and spending time with family—should be encouraged. Future studies should explore not only longitudinal trends but also the effectiveness of tailored interventions across different professional profiles within veterinary medicine. This includes evaluating programs such as structured wellness planning, cognitive-behavioral strategies, and peer-based initiatives. These recommendations must be interpreted in light of the limitations of this study, including the use of non-probabilistic sampling and a cross-sectional design, which restricts causal inference. Longitudinal and interventional designs are required to validate and refine the strategies proposed in this study.

## Figures and Tables

**Table 1:** Occupational and career characteristics of veterinary professionals (n = 1992).

Characteristics	n	Percentage
**Professional profile**		
Monthly income (USD) ^a^		
Up to 640	722	36.2%
641–1165	539	27.1%
1166–2330	354	17.8%
2331+	168	8.4%
I prefer not to answer	209	10.5%
Current position		
Employee at a veterinary clinic	739	37.1%
Owner or co-owner	558	28.0%
Surrogate veterinary	252	12.7%
Academic position (professor, researcher, etc.)	157	7.9%
Non-executive role in the industry	100	5.0%
Consultant	93	4.7%
Other	93	4.7%
Time from graduation (years)		
0–5	748	37.6%
6–10	403	20.2%
11–20	515	25.9%
21+	326	16.4%
**Work conditions**		
Current workload		
I’m working more hours than I would like to	1000	50.2%
I’m working less hours than I would like to	259	13.0%
I’m satisfied with the number of hours I’m working	637	32.0%
I prefer not to answer	96	4.8%
In addition to your regular job, do you have a second job?		
Yes	772	38.8%
No	1172	58.8%
I prefer not to answer	48	2.4%
**Career satisfaction**		
Would you recommend a veterinary career?		
Yes	774	38.9%
No	1126	56.5%
I prefer not to answer	92	4.6%
Do you regret becoming a veterinarian?		
Yes	542	27.2%
No	1368	68.7%
I prefer not to answer	82	4.1%

^a^ Brazilian minimum wage in 2022: US$ 640.

**Table 2:** Multiple logistic regression models for psychological distress (K6) and well-being (PWBI) according to sociodemographic and occupational factors (n = 1992).

Predictors	Psychological Distress (K6)		Well-Being (PWBI)	
	OR (95% CI)	*p*	OR (95% CI)	*p*
**Sociodemographic factors**				
Age	1.00 (0.98–1.03)	0.747	**1.03 (1.01–1.05)**	**0.025**
Gender (ref: Female)	1.25 (0.94–1.65)	0.103	**0.48 (0.37–0.62)**	**<0.001**
Marital status (ref: Single)				
Married	**0.74 (0.59–0.94)**	**0.013**	1.13 (0.88–1.52)	0.285
Separated	1.46 (0.92–2.33)	0.103	**0.86 (1.16–3.26)**	**0.011**
Widower	1.03 (0.20–5.22)	0.979	3.22 (0.31–5.21)	0.738
Parental status (ref: No children)	1.04 (0.81–1.33)	0.755	0.90 (0.69–1.16)	0.404
**Occupational factors**				
Income	**0.85 (0.75–0.98)**	**0.022**	1.09	0.223
Time since graduation	**0.98 (0.95–0.99)**	**0.038**	1.02 (1.00–1.05)	0.053
Workload satisfaction (ref: Satisfied)				
Less than desired	1.26 (0.92–1.73)	0.148	0.80 (0.59–1.10)	0.174
More than desired	**1.74 (1.37–2.21)**	**<0.001**	**0.31 (0.24–0.40)**	**<0.001**
Second job (ref: No)	0.98 (0.80–1.21)	0.878	**1.30 (1.03–1.64)**	**0.030**
**Career satisfaction**				
Recommend career (ref: Yes)	**1.84 (1.44–2.35)**	**<0.001**	**0.42 (0.32–0.53)**	**<0.001**
Regret career (ref: No)	**2.53 (2.00–3.20)**	**<0.001**	**0.54 (0.39–0.74)**	**<0.001**
**Model fit indices**				
K6: χ^2^ = 302.97, *p* < 0.001; Nagelkerke R^2^ = 0.196; Hosmer–Lemeshow test *p* = 0.962				
PWBI: χ^2^ = 468.21, *p* < 0.001; Nagelkerke R^2^ = 0.300; Hosmer–Lemeshow test *p* = 0.386				

Note. OR: odds ratio; CI: confidence interval. Coding: K6 (0 = no distress, 1 = distress); PWBI (0 = impaired well-being, 1 = preserved well-being). Bold values indicate statistically significant results at *p* < 0.05.

**Table 3:** Multiple logistic regression models for psychological distress (K6) and well-being (PWBI) according to coping strategies (n = 1992).

Coping Strategies	Psychological Distress (K6)		Well-Being (PWBI)	
	OR (95% CI)	*p*	OR (95% CI)	*p*
Physical exercise	0.92 (0.83–1.02)	0.119	1.06 (0.95–1.19)	0.281
Yoga practice	1.20 (0.99–1.45)	0.061	**0.79 (0.66–0.94)**	**0.010**
Socialize with friends	**0.82** (**0.71–0.95)**	**0.006**	**1.26 (1.08–1.47)**	**0.003**
Meditate	0.89 (0.77–1.03)	0.119	1.11 (0.97–1.26)	0.130
Read for pleasure	0.94 (0.84–1.06)	0.315	**1.17 (1.04–1.31)**	**0.009**
Travel for pleasure	**0.67 (0.58–0.78)**	**<0.001**	**1.36 (1.17–1.57)**	**<0.001**
Volunteer	**0.86 (0.76–0.97)**	**0.013**	1.07 (0.95–1.21)	0.241
Spend time on a hobby	**0.81 (0.71–0.92)**	**<0.001**	**1.23 (1.08–1.41)**	**0.002**
Spend time with family	**0.69 (0.60–0.80)**	**<0.001**	**1.83 (1.54–2.18)**	**<0.001**
Sleep at least 8 h a night	**0.80 (0.72–0.89)**	**<0.001**	**1.36 (1.21–1.53)**	**<0.001**
**Model fit indices**				
K6: χ^2^ = 334.35, *p* < 0.001; Nagelkerke R^2^ = 0.215; Hosmer–Lemeshow test *p* = 0.383				
PWBI: χ^2^ = 366.20, *p* < 0.001; Nagelkerke R^2^ = 0.241; Hosmer–Lemeshow test *p* = 0.282				

Note. OR: odds ratio; CI: confidence interval. Coding: K6 (0 = no distress, 1 = distress); PWBI (0 = impaired well-being, 1 = preserved well-being). Bold values indicate statistically significant results at *p* < 0.05.

## Data Availability

The original contributions of this study are included in this article. Further inquiries can be directed to the corresponding author.
